# Transluminal endoscopic step-up approach versus minimally invasive surgical step-up approach in patients with infected necrotising pancreatitis (TENSION trial): design and rationale of a randomised controlled multicenter trial [ISRCTN09186711]

**DOI:** 10.1186/1471-230X-13-161

**Published:** 2013-11-25

**Authors:** Sandra van Brunschot, Janneke van Grinsven, Rogier P Voermans, Olaf J Bakker, Marc GH Besselink, Marja A Boermeester, Thomas L Bollen, Koop Bosscha, Stefan A Bouwense, Marco J Bruno, Vincent C Cappendijk, Esther C Consten, Cornelis H Dejong, Marcel GW Dijkgraaf, Casper H van Eijck, G Willemien Erkelens, Harry van Goor, Mohammed Hadithi, Jan-Willem Haveman, Sijbrand H Hofker, Jeroen JM Jansen, Johan S Laméris, Krijn P van Lienden, Eric R Manusama, Maarten A Meijssen, Chris J Mulder, Vincent B Nieuwenhuis, Jan-Werner Poley, Rogier J de Ridder, Camiel Rosman, Alexander F Schaapherder, Joris J Scheepers, Erik J Schoon, Tom Seerden, BW Marcel Spanier, Jan Willem A Straathof, Robin Timmer, Niels G Venneman, Frank P Vleggaar, Ben J Witteman, Hein G Gooszen, Hjalmar C van Santvoort, Paul Fockens

**Affiliations:** 1 Department of Gastroenterology and Hepatology, University of Amsterdam, Amsterdam, The Netherlands; 2 Department of OR/Evidence Based Surgery, Radboud University Nijmegen Medical Centre, Nijmegen, The Netherlands; 3 Department of Surgery, University Medical Center Utrecht, Utrecht, The Netherlands; 4 Department of Surgery, University of Amsterdam, Amsterdam, The Netherlands; 5 Department of Radiology, St Antonius Hospital, Nieuwegein, The Netherlands; 6 Department of Surgery, Jeroen Bosch Hospital, ‘s-Hertogenbosch, The Netherlands; 7 Department of Surgery, Radboud University Nijmegen Medical Centre, Nijmegen, The Netherlands; 8 Department of Gastroenterology, Erasmus MC, University Medical Center, Rotterdam, The Netherlands; 9 Department of Radiology, Jeroen Bosch Hospital, ‘s-Hertogenbosch, The Netherlands; 10 Department of Surgery, Meander Medical Center, Amersfoort, The Netherlands; 11 Department of Surgery and NUTRIM School for Nutrition, Toxicology and Metabolism, Maastricht University Medical Center, Maastricht, The Netherlands; 12 Clinical Research Unit, Academic Medical Center, University of Amsterdam, Amsterdam, The Netherlands; 13 Department of Surgery, Erasmus MC, University Medical Center, Rotterdam, The Netherlands; 14 Department of Gastroenterology, Gelre Hospital, Apeldoorn, The Netherlands; 15 Department of Gastroenterology, Maasstad Hospital, Rotterdam, The Netherlands; 16 Department of Surgery, University Medical Center Groningen, Groningen, The Netherlands; 17 Department of Gastroenterology, Onze Lieve Vrouwe Gasthuis, Amsterdam, The Netherlands; 18 Department of Radiology, University of Amsterdam, Amsterdam, The Netherlands; 19 Department of Surgery, Medical Center Leeuwarden, Leeuwarden, The Netherlands; 20 Department of Gastroenterology, Isala Clinics, Zwolle, The Netherlands; 21 Department of Gastroenterology, VU Medical Center, Amsterdam, The Netherlands; 22 Department of Surgery, Isala Clinics, Zwolle, The Netherlands; 23 Department of Gastroenterology, Maastricht University Medical Center, Maastricht, The Netherlands; 24 Department of Surgery, Canisius-Wilhelmina Hospital, Nijmegen, The Netherlands; 25 Department of Surgery, Leiden University Medical Center, Leiden, The Netherlands; 26 Department of Surgery, Reinier de Graaf Group, Delft, The Netherlands; 27 Department of Gastroenterology, Catharina Hospital, Eindhoven, The Netherlands; 28 Department of Gastroenterology, Amphia Hospital, Breda, The Netherlands; 29 Department of Gastroenterology, Rijnstate Hospital, Arnhem, The Netherlands; 30 Department of Gastroenterology, Máxima Medical Center, Veldhoven, The Netherlands; 31 Department of Gastroenterology, St Antonius Hospital, Nieuwegein, The Netherlands; 32 Department of Surgery, Medisch Spectrum Twente, Enschede, The Netherlands; 33 Department of Gastroenterology and Hepatology, University Medical Center Utrecht, Utrecht, The Netherlands; 34 Department of Gastroenterology, Hospital Gelderse Vallei, Ede, The Netherlands

**Keywords:** Acute pancreatitis, Necrotising, Treatment, Drainage, Trial, Endoscopy, Minimally invasive, Surgery, Necrosectomy, Pancreas

## Abstract

**Background:**

Infected necrotising pancreatitis is a potentially lethal disease that nearly always requires intervention. Traditionally, primary open necrosectomy has been the treatment of choice. In recent years, the surgical step-up approach, consisting of percutaneous catheter drainage followed, if necessary, by (minimally invasive) surgical necrosectomy has become the standard of care. A promising minimally invasive alternative is the endoscopic transluminal step-up approach. This approach consists of endoscopic transluminal drainage followed, if necessary, by endoscopic transluminal necrosectomy. We hypothesise that the less invasive endoscopic step-up approach is superior to the surgical step-up approach in terms of clinical and economic outcomes.

**Methods/Design:**

The TENSION trial is a randomised controlled, parallel-group superiority multicenter trial. Patients with (suspected) infected necrotising pancreatitis with an indication for intervention and in whom both treatment modalities are deemed possible, will be randomised to either an endoscopic transluminal or a surgical step-up approach. During a 4 year study period, 98 patients will be enrolled from 24 hospitals of the Dutch Pancreatitis Study Group. The primary endpoint is a composite of death and major complications within 6 months following randomisation. Secondary endpoints include complications such as pancreaticocutaneous fistula, exocrine or endocrine pancreatic insufficiency, need for additional radiological, endoscopic or surgical intervention, the need for necrosectomy after drainage, the number of (re-)interventions, quality of life, and total direct and indirect costs.

**Discussion:**

The TENSION trial will answer the question whether an endoscopic step-up approach reduces the combined primary endpoint of death and major complications, as well as hospital stay and related costs compared with a surgical step-up approach in patients with infected necrotising pancreatitis.

## Background

Acute pancreatitis is a common and potentially lethal disease. About 20% of patients develop necrosis of the pancreatic parenchyma and/or extrapancreatic fat tissue [1,2]. Necrotising pancreatitis is associated with pancreatic and/or peripancreatic collections with fluid and necrosis. As long as these collections remain sterile, treatment is generally conservative. However, in one third of patients infection of necrosis occurs. Infected necrosis is associated with a mortality rate of around 30% (range 12-39%) [1,3-5] and is virtually always an indication for invasive treatment.

The current treatment of choice is a surgical step up approach [6]. This approach consists of percutaneous catheter drainage, if necessary, followed by surgical necrosectomy. A recent randomised controlled trial demonstrated that this approach reduces death and major complications from 69% to 40% compared with primary open necrosectomy [2]. Furthermore, catheter drainage obviates the need of surgical necrosectomy and associated complications in 35% of patients [2]. Although this trial did not compare minimally invasive necrosectomy to open necrosectomy it did show superiority of the ‘surgical step-up approach’ to primary open necrosectomy. A promising alternative gaining worldwide popularity is endoscopic transluminal drainage and necrosectomy. These procedures can be performed under procedural sedation, thereby avoiding general anaesthesia [7]. Furthermore, abdominal wall incision with its related surgical stress and complications such as incisional hernia, pancreatic fistula and wound infection, are evaded. The endoscopic technique can also be applied in a step-up fashion, consisting of endoscopic transluminal drainage (ETD) followed, if necessary, by endoscopic transluminal necrosectomy (ETN). In recent years, several observational cohort studies have been published reporting the endoscopic treatment of infected necrosis. A small randomised pilot trial has shown that endoscopic necrosectomy is feasible and reduces the inflammatory response, and possibly complications such as new onset organ failure compared with surgical necrosectomy in these often already critically ill patients [8]. Although initial results seem promising, a randomised controlled trial with clinically relevant and applicable endpoints is needed to compare the endoscopic and surgical step-up approach in order to reach a sound evidence-based conclusion about the superiority of either treatment modality.

## Methods

### Study objectives

The primary aim of this study is to investigate whether an endoscopic step-up approach will reduce the combined primary endpoint of death and major complications, as well as the secondary endpoints, hospital stay and costs, as compared to a surgical step-up approach in patients with infected necrotising pancreatitis.

A secondary aim is to investigate whether endoscopic transluminal drainage (ETD) is effective in preventing necrosectomy.

### Design

The TENSION trial is a randomised controlled, parallel-group, superiority multicenter trial. Patients will be randomly allocated using an internet-based randomisation program (Academic Medical Center) to the endoscopic or surgical step-up approach. Patients with (suspected) infected necrosis are eligible for randomisation. The trial is registered in the ISRCTN register (ISRCTN09186711).

### Participating centers

Twenty-four hospitals of the Dutch Pancreatitis Study Group (DPSG), including all Dutch university medical centers, participate in the TENSION trial and will enrol patients (see Appendix for a list of all participating centers). Interventions will only take place in centers with sufficient expertise and after the indication for intervention is supported by an online expert panel.

### Primary endpoint

The primary endpoint is a composite of death or major complications occurring within 6 months following randomisation.

Major complications are defined as new onset (i.e. not present 24 hours before randomisation) organ failure (cardiovascular, pulmonary or renal), bleeding requiring intervention, perforation of a visceral organ requiring intervention, enterocutaneous fistula requiring intervention and incisional hernia (including burst abdomen) (see Table 1 for definitions).

**Table 1:** Primary endpoint: definitions

**Event**	**Definition**
Organ failure	Organ failure is defined as:
	• Cardiovascular: systolic blood pressure < 90 mmHg despite adequate fluid resuscitation or need for vasopressor support
	• Pulmonary: PaO_2_ < 60 mmHg despite FiO_2_ 30%, or the need for mechanical ventilation;
	• Renal: serum creatinine > 177 mmol/L after rehydration or need for hemofiltration or hemodialysis;
	Definitions are adapted from the Atlanta classification and the same as previously used in the PANTER trial [2]
New onset organ failure	Organ failure occurring after randomisation and not present 24 hours before randomisation
Multiple organ failure	Failure of 2 or more organ systems on the same day
Enterocutaneous fistula	Enterocutaneous fistula is defined as secretion of fecal material from a percutaneous drain, drainage canal after removal of drains, or from a surgical wound, either from small or large bowel; confirmed by imaging or during surgery [2]
Incisional hernia	Incisional hernia is defined as a full-thickness discontinuity of the abdominal wall and bulging of abdominal contents, with or without obstruction [2]

### Secondary endpoints

•the individual components of the primary endpoint

•pancreaticocutaneous fistula (see Table 2 for definitions)

•exocrine or endocrine pancreatic insufficiency (see Table 2 for definitions)

•biliary strictures

•wound infections (see Table 2 for definitions)

•the need for necrosectomy (either endoscopically or surgically)

•the need for additional radiological, endoscopic or surgical interventions

•number of radiological, endoscopic and surgical (re-)interventions

•total length of intensive care and hospital stay

•quality of life, quality adjusted life year’s (QALY’s, with Short Form 36 and EQ5D)

•costs per patient with poor outcome and costs per QALY

•total direct and indirect medical costs

•total number of cross-over between groups

**Table 2:** Secondary endpoint: definitions

**Event**	**Definition**
Pancreaticocutaneous fistula	Pancreaticocutaneous fistula is defined as output, through a percutaneous drain, drainage canal after removal of drains, or from a surgical wound, of any measurable volume of fluid with an amylase content > 3 times the serum amylase level
Pancreatic insufficiency	• Exocrine insufficiency is defined as an abnormal fecal elastase test or the need for oral pancreatic-enzyme supplementation to treat clinical symptoms of steatorrhea (not present before onset pancreatitis)
	• Endocrine insufficiency is defined as insulin or oral antidiabetic drugs required (not present before onset pancreatitis)
Wound infection [9]	Wound infection is defined as a superficial incisional surgical site infection (SSI) and must meet the following criterion:
	Infection occurs within 30 days after the operative procedure and involves only skin and subcutaneous tissue of the incision and the patient has at least 1 of the following:
	­purulent drainage from the superficial/deep incision but not from the organ/space component of the surgical site
	­organisms isolated from an aseptically obtained culture of fluid or tissue from the superficial incision
	­at least 1 of the following signs or symptoms of infection: pain or tenderness, localized swelling, redness, or heat
	­the superficial incision is deliberately opened by surgeon and is culture positive or not cultured. A culture-negative finding does not meet this criterion
	­an abscess or other evidence of infection involving the deep incision is found on direct examination, during reoperation, or by histopathologic or radiologic examination
	­diagnosis of superficial/deep incisional SSI by the surgeon or attending physician

### Study population

All patients admitted or transferred to one of the 24 participating hospitals of the DPSG with (suspected) infected necrosis and an indication for intervention will be assessed for eligibility. Patients (or their legal representatives) that meet the in- and exclusion criteria will be asked for written informed consent.

### Inclusion criteria

•(Suspected) infected pancreatic and/or extrapancreatic necrosis and an indication for intervention [2,10] (see Table 3 for definitions).

•Both the endoscopic step-up approach and the surgical step-up approach are technically feasible

•Age ≥ 18 years

### Exclusion criteria

•Previous intervention (e.g. surgical, endoscopic or percutaneous) for pancreatic necrosis, extrapancreatic necrosis and/or peripancreatic collections (see Table 3 for definitions)

•Acute flare up of chronic pancreatitis

•Indication for emergency laparotomy because of suspected abdominal compartment syndrome, bowel ischemia, bleeding or perforation of a visceral organ

**Table 3:** Inclusion and exclusion criteria: definitions

**Event**	**Definition**
Pancreatic necrosis	Diffuse or focal area(s) of non-enhancing pancreatic parenchyma as detected on contrast enhanced CT (CECT)
Extrapancreatic necrosis	Persistent peripancreatic fluid collections on CECT in the absence of pancreatic parenchymal non-enhancement
(Suspected) infected necrosis	• Infected necrosis is defined as a positive culture of pancreatic necrosis or extrapancreatic necrosis obtained by fine-needle aspiration (FNA) or the presence of gas in the fluid collection on CECT.
	• Suspected infected necrosis is defined as persistent sepsis or progressive clinical deterioration despite maximal support on the intensive care unit (ICU) in case of pancreatic necrosis or extrapancreatic necrosis, without documentation of infected necrosis and without other causes for infection
Previous intervention	Previous exploratory laparotomy for suspected abdominal compartment syndrome, bleeding or suspected bowel perforation is only allowed if the omental bursa was not opened
MODS	The Multiple Organ Dysfunction Score (MODS) ranges from 0 to 24, with higher scores indicating more severe organ dysfunction
SOFA	Scores on the Sequential Organ Failure Assessment (SOFA) scale range from 0 to 24, with higher scores indicating more severe organ dysfunction

### Randomisation

**Figure 1:**
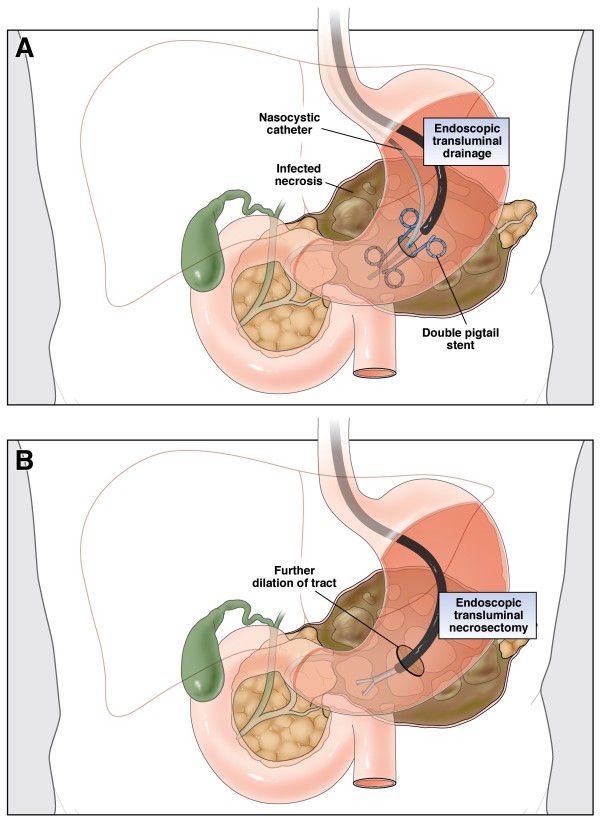
**Endoscopic step-up approach.** Endoscopic step-up approach consisting of endoscopic transluminal drainage (ETD) and endoscopic transluminal necrosectomy (ETN). A large peripancreatic collection containing fluid and necrosis is shown. **(A)** ETD: the collection is punctured through the gastric wall, followed by balloon dilatation of the tract. Two double-pigtail stents and a nasocystic catheter for continuous postoperative irrigation are placed. **(B)** ETN: the cystostomy tract is dilated, the collection is entered with a endoscope, and necrosectomy is performed. (Reprinted from van Brunschot et al. [11]; copyright 2013, with permission from Elsevier).

**Figure 2:**
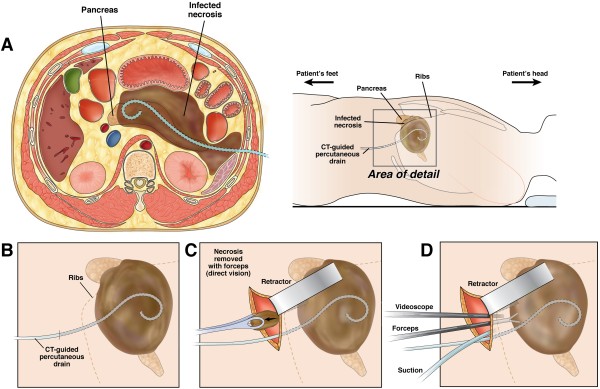
**Surgical step-up approach.** Surgical step-up approach consisting of percutaneous catheter drainage (PCD) and video-assisted retroperitoneal débridement (VARD). **(A)** Cross-sectional image and torso depicting a peripancreatic collection. The preferred route is through the left retroperitoneal space between the kidney, spleen and descending colon. A percutaneous catheter drain is inserted in the collection to mitigate sepsis and postpone or even obviate necrosectomy. The area of detail is shown in **(B)**. **(C)** A 5 cm subcostal incision is made and the percutaneous drain is followed into the collection. The first necrosis is removed under direct vision with a long grasping forceps, followed by further debridement under videoscopic assistance **(D)**. (Reprinted from van Brunschot et al. [11]; copyright 2013, with permission from Elsevier).

**Figure 3:**
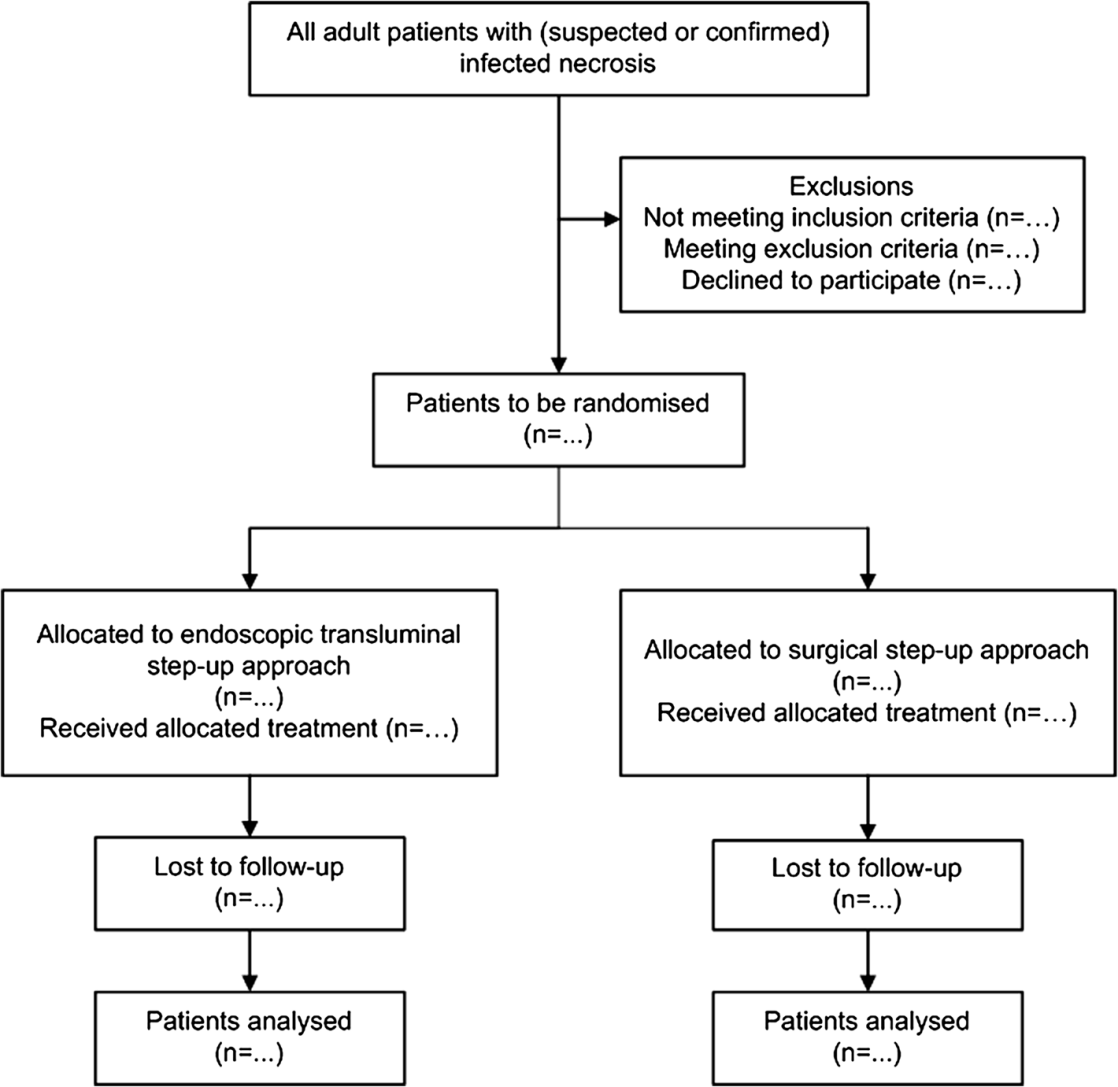
**Flowchart TENSION trial according to CONSORT **[12]**.**

**Figure 4:**
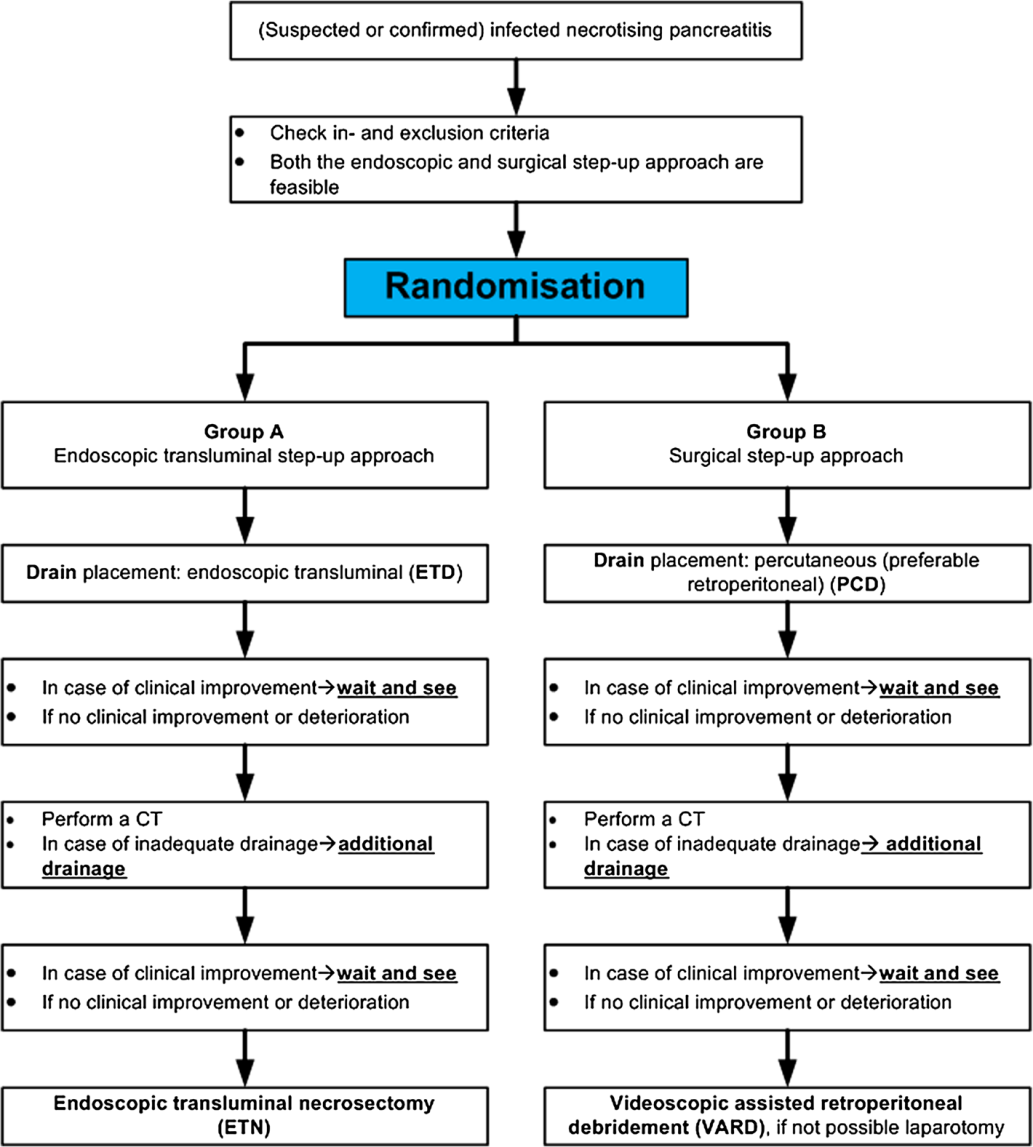
Flowchart treatment protocol TENSION trial.

If a patient with pancreatic and/or extrapancreatic necrosis shows clinical deterioration and has reached the stage to decide on invasive intervention for (suspected) infected necrosis, the Dutch nationwide expert panel is consulted. This panel, consisting of 17 experts (9 surgeons, 4 gastroenterologists and 4 radiologists) is available 24 hours a day, 7 days a week, to assess the indication for intervention, the feasibility of both treatment options and advises on timing of intervention. In general, intervention is delayed to a phase of the disease at which necrosis is walled-off, usually 3–4 weeks after onset. A similar expert panel has already proven to be of value during the previous Dutch PANTER and PENGUIN trials [2,10]. After consultation of the expert panel, patients eligible for inclusion are randomly assigned to group A (endoscopic step-up approach, see Figure 1) or B (surgical step-up approach, see Figure 2) as shown in the flowcharts (Figures 3 and 4). Randomisation is performed by the study coordinator using an internet-based randomisation program (Academic Medical Center) ensuring allocation concealment between groups. Randomisation is stratified according to hospital.

### Treatment protocol

#### Group A (Endoscopic transluminal step-up approach)

##### Step 1: endoscopic transluminal drainage (ETD)

Using procedural sedation, with either i.v. administration of midazolam and fentanyl or propofol, endoscopic ultrasound guided transluminal drainage of the peripancreatic collection is performed (see Figure 1). Two 7 Fr double pigtail stents and a naso-cystic catheter are inserted into the collection. The latter will be used for continuous flushing with 1 liter saline/24 hours. In case of clinical improvement (see criteria below), subsequent necrosectomy is avoided. In case a patient does not improve after 72 hours and the collection is deemed inadequately drained as observed on repeat CECT, additional drainage is performed. If re-drainage is not indicated (drains are well positioned in the fluid cavity), clinically unsuccessful or impossible, the patient will proceed to step 2.

##### Step 2: endoscopic transluminal necrosectomy (ETN)

The cystogastrostomy is dilated up to 18 mm and the cavity is entered with a therapeutic gastroscope to perform necrosectomy under direct endoscopic vision (see Figure 1). The procedure is completed when most loose adherent necrotic tissue is removed. Again two 7 Fr double pigtail stents and a naso-cystic catheter for continuous lavage will be inserted into the collection. The procedure is repeated in case there is no clinical improvement within 72 hours.

#### Group B (Surgical step-up approach)

This approach is similar to the step-up approach used in the PANTER trial [2,10].

##### Step 1: percutaneous catheter drainage (PCD)

A percutaneous 14 to 20 French drain is placed in the peripancreatic collection under guidance of CT or ultrasound (see Figure 2). The preferred route is through the left retroperitoneum, thereby facilitating video-assisted retroperitoneal débridement (VARD) [13] at a later stage if needed. If this is not possible, trans-peritoneal drainage is performed. Drains are kept open by flushing with 50 ml saline three times daily. In case of clinical improvement, the further effect of drainage is awaited. If a patient is not improving and a collection is deemed inadequately drained on repeat CECT after 72 hours, additional drainage is performed. If this is not possible, or if a second drainage is clinically unsuccessful (see criteria for clinical improvement below) the patient will proceed to step 2.

##### Step 2: video-assisted retroperitoneal debridement (VARD, if not possible laparotomy)

VARD is a drain-guided, minimally invasive retroperitoneal procedure requiring a 5 cm flank incision according to the previously published technique [13,14] (see Figure 2). Using the retroperitoneal drain for guidance, the collection is entered and only loosely adherent necrosis is removed under video-assistance. At the end of the procedure two large bore surgical drains are inserted. A continuous post-operative lavage system (building up to 10 litres saline per 24 hrs) is installed. In case of absence of clinical improvement (see criteria below) and repeated CECT shows foci of potentially inadequate drainage, VARD is repeated. If VARD is technically not feasible, debridement by laparotomy is performed.

If drainage technically fails in the endoscopic group a PCD is placed. In case of clinical or technical failure of PCD, surgical necrosectomy is performed. Both approaches are performed, according to a strict protocol, only in participating centers with documented expertise and, if necessary, under supervision of an expert. Sufficient expertise is defined as having performed at least ten independent VARD procedures or ten independent endoscopic transluminal drainage procedures and more than five endoscopic transluminal necrosectomies. In case of insufficient local experience, the patient is transferred to a tertiary referral center with sufficient experience.

#### Criteria for clinical improvement

Criteria similar to the PANTER trial are used to define clinical improvement, failure and to decide to go to the next step [2,10]. Each step is evaluated 72 hours after intervention and considered successful in case of clinical improvement. Clinical improvement is defined as: improved function of at least two organ systems (i.e. circulatory, pulmonary, or renal) or improvement of two out of three parameters of infection (i.e. C-reactive protein, leucocytes, or temperature). Clinical failure is defined as the absence of clinical improvement or in case of clinical deterioration. If there is, at evaluation or any moment thereafter, clinical failure the next step or next necrosectomy is performed. Deterioration by other infectious causes than infected necrosis (e.g. a urinary tract infection) is excluded.

#### General treatment regimen

All patients receive enteral nutrition. If oral feeding is not tolerated or insufficient, a nasojejunal feeding tube is introduced and enteral feeding is started. If the required caloric intake cannot be reached via the enteral route, the patient will receive (additional) parenteral nutrition. All patients with (suspected) infected necrosis will receive broad-spectrum antibiotic therapy according to institutional protocols. Antibiotic treatment is tailored based on blood cultures and culture from material collected during drainage or surgical procedures. If cultures remain negative, antibiotic treatment is stopped. Before retraction or removal of a percutaneous drain or the pigtail stents the remaining cavity is visualized.

### Data collection

Clinical data with regard to baseline characteristics and outcome are collected during hospital admission using a standardised case record form (CRF). An independent monitor checks all endpoints and at least 10% of the CRF data with on-site source data.

### Follow-up

Patients are observed during their hospital stay. Outpatient follow-up visits are scheduled at the discretion of the responsible physician, but always 3 and 6 months after randomisation and 3 and 6 months after discharge. During these visits all patients will undergo a routine CECT, exocrine and endocrine pancreatic function tests (i.e. blood glucose measurements and fecal elastase test), and receive a combined questionnaire (SF-36 [15], EQ-5D [16], and Health and Labour [17]).

### Safety

At regular intervals, an independent data safety and monitoring committee (DSMC) will evaluate the progress of the trial and examine the unblinded safety variables [18]. All physicians involved in the study will repetitively be asked to report any potential adverse events. These events will be listed and discussed with the DSMC. All possible adverse events will also be reported to the Central Committee on Research involving Human Subjects and the institutional review board (IRB). Adverse events are defined as ‘any undesirable experience occurring to a subject during a clinical trial, whether or not considered related to the intervention’. The outcome of the meeting of the DSMC will be discussed with the trial steering committee and sent to the IRB.

### Ethics

The study is performed in accordance with the declaration of Helsinki and the Dutch Medical Research Involving Human Subjects Act. The IRB of the Academic Medical Centre Amsterdam approved the protocol on the 31th of January 2011. Secondary approval was sought from all local ethics committees. Informed consent will be obtained from each participating patient in oral and written form prior to randomisation. The TENSION trial is registered in the ISRCTN register with identification number ISRCTN09186711. After approval of the protocol, no amendments on study design were made.

### Statistical aspects

#### Sample size calculation

The TENSION trial is a superiority trial, hypothesizing a reduction in the primary endpoint in favour of the endoscopic step-up approach. Combined results of published non-randomised studies on ETN were used to calculate the sample size. These cohort studies show that ETN results in a combined death and major complication rate of 17% [19-24]. The previous randomised PANTER trial showed a combined death and major complication rate of 40% for the surgical step-up approach [2]. Furthermore, in the surgical group an incisional hernia rate of 7% was seen [2]. Assuming that some patients will develop an incisional hernia in the surgical group without having another primary endpoint, the prevalence of death and major complications in this group, including incisional hernias is estimated to be 43%. Therefore, an absolute reduction in primary endpoint of 26% (from 43 to 17%) is anticipated. With a 2-sided 5% alpha, power of 80%, and 2% loss to follow-up, the sample size was set at 98 patients.

#### Descriptive statistics

For dichotomous data, frequencies will be presented. Continuous data will be presented as mean and standard deviation or median and interquartile range. Baseline criteria are: age, sex, body mass index, aetiology of pancreatitis, co-morbidity, American Society of Anaesthesiologist’s (ASA) classification, CT severity index, extent of pancreatic necrosis, disease severity (SIRS, ICU admission, single or multiple organ failure), Acute Physiology and Chronic Health Evaluation (APACHE) ll score, Imrie score, MODS (Table 3), SOFA score (Table 3), C-reactive protein, time from onset of symptoms to randomisation, tertiary referral, and confirmed infected necrosis (bacterial culture of first intervention).

#### Analyses

There will be a blinded outcome assessment after the last patient completed follow-up for all primary and secondary endpoints. Both intention-to-treat and per-protocol analyses will be performed. In intention-to-treat analysis, all patients are analysed according to their initially assigned study arm regardless of adherence to study protocol, which is the primary analysis of the study. Occurrences of the primary and secondary endpoints are compared between treatment groups. Comparison of the primary endpoint will be expressed in terms of a relative risk and corresponding 95% confidence intervals. A two-tailed P < 0.05 is considered statistically significant. Subsequent analyses are directed at secondary endpoints. Predefined subgroup analysis will be performed for patients with and without (multiple) organ failure (see Table 1 for definitions) before randomisation, institution and time between onset of symptoms and randomisation (<28 or >28 days). A formal test of interaction in a logistic-regression model is used to assess whether treatment effects differ significantly between subgroups. In case of skewed randomisation (i.e. statistically significant differences in baseline variables), an adjusted analysis will be performed using multivariable logistic regression.

#### Additional analyses

Direct and indirect medical and non-medical costs of both treatment strategies for the follow-up period of 6 months after randomisation will be compared. All costs will be estimated based on the actual input in terms of resource use (i.e. interventions, diagnostic procedures, hospital and ICU stay), personnel, medication, visits to healthcare providers, private household assistance, and indirect costs from loss of productivity due to sick leave (assessed with the Health and Labour questionnaire). Total costs per patient are calculated by summing direct medical costs, direct nonmedical costs, and indirect costs and subsequently compared between groups. Furthermore, the impact of differences in complications on the quality of life is measured by a generic quality of life questionnaire, the SF-36. In addition to this quality of life questionnaire, the EQ-5D is completed which screens for the presence and severity of problems with mobility, self-care, daily activities, pain/complaints and mood.

#### Premature termination of the study

No formal interim-analysis is planned. To guarantee patient’s safety throughout the study, the DSMC will perform regular safety analyses. When harm (higher incidence of SAE’s in one group) occurs, the DSMC will discuss potential stopping of the trial prematurely with the trial steering committee. Since this is the first randomised trial comparing both approaches, and hence all data arising from this trial, regardless of its outcome, will influence treatment policy worldwide, the trial will not be stopped for futility.

## Discussion

The TENSION trial is designed to answer the question whether an endoscopic step-up approach will lead to a reduction of death and major complications compared to a surgical step-up approach in patients with (suspected) infected necrosis. The TENSION trial will also investigate whether pancreatic fistula, exocrine or endocrine pancreatic insufficiency, length of ICU and hospital stay, quality of life and costs are reduced by the endoscopic step-up approach.

In recent years, minimally invasive approaches are gradually replacing (primary) open necrosectomy. Minimally invasive approaches aim at minimizing surgical stress and have proven to reduce complications. In the PANTER trial, the surgical step-up approach reduced the combined death and major complication rate from 69% to 40% [2]. Furthermore, the PANTER trial showed that 35% of patients with infected necrotising pancreatitis achieve complete recovery after percutaneous drainage only, without the need for surgical debridement. Although the combined death and major complication rate is still high, the surgical step-up approach should at present be considered as the current standard of care worldwide [2]. Drainage is based on the hypothesis that alleviating pressure of an infected collection may improve the patient’s clinical condition and thereby leaving the necrotic tissue to be dealt with by the patient’s own immune system. Endoscopic transluminal drainage can be applied according to the same rationale. Therefore, we chose to institute the step-up approach in both study arms. Due to the large differences in treatments between both groups, blinding is not feasible. To partially compensate for this, outcome assessment is blinded.

In the TENSION trial only patients with (suspected) infected necrosis are included since the main indication for intervention in necrotising pancreatitis is nowadays considered to be infected necrosis [25-28]. Patients with sterile necrosis can often be successfully managed conservatively (i.e. without any form of intervention) [28-30].

A composite endpoint of death and major complications was chosen because a study powered to demonstrate a clinically relevant difference in death alone would require an unrealistic large sample size of over 2000 patients. In addition, previous studies have shown that major complications have high impact in terms of quality of life in patients with necrotising pancreatitis [2,8].

A potential limitation of the endoscopic approach is that periprocedural complications (e.g. perforation or bleeding) may be more difficult to manage when compared to periprocedural complications occurring during surgical necrosectomy. A systematic review and randomised trial have suggested that endoscopic treatment of infected necrosis is feasible and associated with lower or comparable complication rates than surgery [7,8]. Furthermore, endoscopic drainage and necrosectomy are advanced interventions that not only require the expertise from an interventional endoscopist, but also the dedicated involvement of interventional radiologists and pancreatic surgeons to manage potential complications. For this reason the endoscopic interventions in the TENSION trial will only be performed in expert centers with multidisciplinary expertise.

## Conclusion

The TENSION trial is a randomised controlled multicenter trial designed to show a reduction in the composite primary endpoint of death and major complications, as well in hospital stay and costs following an endoscopic transluminal step up approach compared with a surgical step up approach in patients with infected necrotising pancreatitis.

## Appendix

### TENSION committee members

#### Steering committee

S. van Brunschot, MD, Dept. of Gastroenterology and Hepatology, Academic Medical Center, University of Amsterdam and dept. of OR/Evidence Based Surgery, Radboud University Nijmegen Medical Center

H.C. van Santvoort, MD PhD, Dept. of Surgery, University Medical Center Utrecht

M.G.H. Besselink, MD PhD, Dept. of Surgery, Academic Medical Center

O.J. Bakker, MD PhD, Dept. of Surgery, University Medical Center Utrecht

P. Fockens, MD PhD, Dept. of Gastroenterology and Hepatology, Academic Medical Center, University of Amsterdam *(chair)*

H.G. Gooszen, MD PhD, Dept. of OR/Evidence Based Surgery, Radboud University Nijmegen Medical Center

M.A. Boermeester, MD PhD, Dept. of Surgery, Academic Medical Center

M.J. Bruno, MD PhD, Dept. of Gastroenterology, Erasmus MC, University Medical Center

C.H.C. Dejong, MD PhD, Dept. of Surgery and NUTRIM School for Nutrition, Toxicology and Metabolism, Maastricht University Medical Center

R. Timmer, MD PhD, Dept. of Gastroenterology, St Antonius Hospital

B.J.M. Witteman, MD PhD, Dept. of Gastroenterology, Gelderse Vallei Hospital

#### Expert panel

M.A. Boermeester, MD PhD, Dept. of Surgery, Academic Medical Center, Amsterdam

T.L. Bollen, MD, Dept. of Radiology, St Antonius Hospital, Nieuwegein

M. Bruno, MD PhD, Dept. of Gastroenterology, Erasmus MC, University Medical Center, Rotterdam

V.C. Cappendijk, MD, Dept. of Radiology, Jeroen Bosch Hospital, 's-Hertogenbosch

C.H.C. Dejong, MD PhD, Dept. of Surgery and NUTRIM School for Nutrition, Toxicology and Metabolism, Maastricht University Medical Center, Maastricht

C. van Eijck, MD PhD, Dept. of Surgery, Erasmus MC, University Medical Center, Rotterdam

P. Fockens, MD PhD, Dept. of Gastroenterology and Hepatology, Academic Medical Center, University of Amsterdam, Amsterdam

H. van Goor, MD PhD, Dept. Of Surgery, Radboud University Nijmegen Medical Center, Nijmegen

H.G. Gooszen, MD PhD, Dept. of OR/Evidence Based Surgery, Radboud University Nijmegen Medical Center, Nijmegen

J.W. Haveman, MD PhD, Dept. of Surgery, University Medical Center Groningen, Groningen

H.S. Hofker, MD PhD, Dept. of Surgery, University Medical Center Groningen, Groningen

J.S. Laméris, MD PhD, Dept. of Radiology, Academic Medical Center, Amsterdam

K.P. van Lienden, MD PhD, Dept. of Radiology, Academic Medical Center, Amsterdam

V.B. Nieuwenhuijs, MD PhD, Dept. of Surgery, Isala Clinics, Zwolle

J.W. Poley, MD PhD, Dept. of Gastroenterology, Erasmus MC, University Medical Center, Rotterdam

A.F.M. Schaapherder, MD PhD, Dept. of Surgery, Leiden University Medical Center, Leiden

R. Timmer, MD PhD, Dept. of Gatroenterology, St Antonius Hospital, Nieuwegein

#### Data and Safety Monitoring Committee

J.G.P. Tijssen, MD PhD, Dept. of Clinical Epidemiology, Academic Medical Center, Amsterdam (chair)

J.F. Lange, MD PhD, Dept. of Surgery, Erasmus MC, University Medical Center, Rotterdam

H.J. Bonjer, MD PhD, Dept. of Surgery, VU Medical Center, Amsterdam

J. Stoker, MD PhD, Dept. of Radiology, Academic Medical Center, Amsterdam

A.A.M. Masclee, MD PhD, Dept. of Gastroenterology, Maastricht University Medical Center, Maastricht

#### Independent physician

K.M.A.J. Tytgat, MD PhD, Dept. of Gastroenterology, Academic Medical Center, Amsterdam

### Clinical centers and principal investigators (all in the Netherlands, alphabetical order)

1. Academic Medical Center, PO 22660, 1100 DD Amsterdam; P Fockens, MD PhD, Dept. of Gastroenterology and Hepatology;

2. Amphia Hospital Breda, PO 90158, 4800 RK Breda; T Seerden, MD PhD, Dept. of Gastroenterology;

3. Canisius-Wilhelmina Hospital, PO 9015, 6500 GS Nijmegen; C Rosman, MD PhD, Dept. of Surgery;

4. Catharina Hospital, PO 1350, 5623 EJ Eindhoven; EJ Schoon, MD PhD, Dept. of Gastroenterology;

5. Erasmus Medical Center, PO 2040, 3000 CA Rotterdam; MJ Bruno, MD PhD, Dept. of Gastroenterology;

6. Gelre Hospital, PO 9014, 7300 DS Apeldoorn; GW Erkelens, MD, Dept. of Gastroenterology;

7. Hospital Gelderse Vallei, PO 9025, 6710 HN Ede; B Witteman, MD PhD, Dept. of Gastroenterology;

8. Isala Clinics Zwolle, PO 10400, 8000 GK Zwolle; MAC Meijssen, MD PhD, Dept. Of Gastroenterology;

9. Jeroen Bosch Hospital, PO 90153, 5200 ME ‘s-Hertogenbosch; K Bosscha, MD PhD, Dept. of Surgery;

10. Leiden University Medical Center, PO 9600, 2300 RC Leiden; AFM Schaapherder, MD PhD, Dept. of Surgery;

11. Maasstad Hospital Rotterdam, PO 9100, 3007 AC Rotterdam; H Hadithi, MD, Dept. of Gastroenterology;

12. Maastricht University Medical Center, PO 5800, 6202 AZ Maastricht; RJJ de Ridder, MD PhD, Dept. Of Gastroenterology;

13. Máxima Medical Center Veldhoven, PO 7777, 5500 MB Veldhoven; JWA Straathof, MD PhD, Dept. of Gastroenterology;

14. Meander Medical Center, PO 1502, 3800 BM, Amersfoort; EC Consten, MD PhD, Dept. of Surgery;

15. Medical Center Leeuwarden, PO 888, 8901 BR Leeuwarden; ER Manusama, MD PhD, Dept. of Surgery;

16. Medical Spectre Twente, PO 50000, 7500 KA Enschede; NG Venneman, MD PhD, Dept. of Gastroenterology;

17. OLVG Amsterdam, PO 95500, 1090 HM Amsterdam; JM Jansen, MD PhD, Dept. of Gastroenterology;

18. Radboud University Nijmegen Medical Center, PO 9101, 6500 HB Nijmegen; H van Goor, MD PhD, Dept. of Surgery;

19. RdGG Delft, PO 5011, 2600 GA Delft; JJG Scheepers, MD, dept. of Surgery;

20. Rijnstate Hospital, PO 9555, 6800 TA Arnhem; BWM Spanier, MD PhD, Dept. of Gastroenterology;

21. St Antonius Hospital, PO 2500, 3430 EM Nieuwegein; R Timmer, MD PhD, Dept. of Gastroenterology;

22. University Medical Center Groningen, PO 30001, 9700 RB Groningen; HS Hofker, MD PhD, Dept. of Surgery;

23. University Medical Center Utrecht, PO 85500, 3508 GA Utrecht; FP Vleggaar, MD PhD, Dept. of Gastroenterology and Hepatology;

24. VU Medical Center Amsterdam, PO 7057, 1007 MB Amsterdam; CJ Mulder, MD PhD, Dept. of Gastroenterology.

### Key staff at coordinating centers

Academic Medical Center Amsterdam: P Fockens (principal investigator), S van Brunschot (coordinator);

Radboud University Nijmegen Medical Centre: S van Brunschot (coordinator), J van Grinsven (coordinator), H van der Eng (research nurse) and HG Gooszen;

University Medical Center Utrecht: HC van Santvoort

## Abbreviations

CECT: Contrast enhanced computed tomography; CRF: Case record form; DPSG: Dutch pancreatitis study group; DSMC: Data safety monitoring committee; ETD: Endoscopic transluminal drainage; ETN: Endoscopic transluminal necrosectomy; ISRCTN: International standard randomised controlled trial number; PCD: Percutaneous catheter drainage; QALY: Quality adjusted life year; VARD: Video-assisted retroperitoneal debridement.

## Competing interests

The authors declare that they have no competing interests.

## Authors’ contributions

SvB drafted the manuscript. HCvS, JvG, RV, OJB, MGB, HGG and PF co-authored the writing of the manuscript. SvB, HCvS, MGHB, OJB, RPV, MGWD, MAB, MJB, CHCD, RT, BJW, HGG, and PF participated in the design of the study prior to and during several meetings of the Dutch Pancreatitis Study Group. SvB, HCvS, RPV and MGWD performed the sample size calculation. All authors critically assessed the study design or included patients in the study, edited the manuscript and read and approved the final manuscript.

## Pre-publication history

The pre-publication history for this paper can be accessed here:

http://www.biomedcentral.com/1471-230X/13/161/prepub
